# Characterization of Iranian nonaflatoxigenic strains of *Aspergillus flavus* based on microsatellite-primed PCR

**Published:** 2015-01

**Authors:** Mahmoud Houshyarfard, Hamid Rouhani, Mahrokh Falahati-Rastegar, Saeid Malekzadeh-Shafaroudi, Esmat Mahdikhani-Moghaddam

**Affiliations:** 1 Department of Plant Protection, Faculty of Agriculture, Ferdowsi University of Mashhad, Iran; 2 Department of Crop Biotechnology and Breeding, Faculty of Agriculture, Ferdowsi University of Mashhad, Iran

**Keywords:** Aflatoxin, Molecular marker, Inter-simple sequence repeat, Polymorphism

## Abstract

Out of fifty-two Iranian nonaflatoxigenic strains of *Aspergillus flavus*,collected from various substrates (soil and kernel) and sources (peanut, corn and pistachio)*, *fifteen representatives were selected according to their different geographical origins (six provinces: Guilan and Golestan, Ardebil, Fars, Kerman and Semnan) and vegetative compatibility groups (VCGs, IR1 to IR15) for microsatellite-primed PCR analysis. Two inter-simple sequence repeat (ISSR) primers AFMPP and AFM13 were used to determine polymorphism and the relationship among strain isolates. *A. flavus* isolates were identified by their morphologies and their identities were confirmed by PCR amplification using the specific primer pair ITS1 and ITS4. The results revealed variations in the percentages of polymorphisms. In the ISSR analysis, primers AFMPP and AFM13 generated a total of 18 and 23 amplicons among the fungal strains, out of which 12 (66.7%) and 22 (95.7%) were polymorphic, respectively. Cluster analysis of the ISSR data was carried out using 1 D DNA gel image analysis. The two dendrograms obtained through these markers showed six different clusterings of testing nonaflatoxigenic *A. flavus* L strains, but we noticed that some clusters were different in some cases. The microsatellite-primed PCR data revealed that the Iranian nonaflatoxigenic isolates of *A. flavus* were not clustered according to their origins and sources. This study is the first to characterize Iranian nonaflatoxigenic isolates of* A. flavus* using ISSR markers.

## INTRODUCTION

*Aspergillus flavus* Link. ex Fries, a haploid organism found worldwide in a variety of crops, including maize, cottonseed, almond, pistachio and peanut, causes substantial and recurrent worldwide economic liabilities [1, 2]. This filamentous fungus produces aflatoxins (AFLs) B_1_ and B_2_, which are among the most carcinogenic, acutely hepatotoxic and immunosuppressive compounds found in nature [3-5]. Recent efforts to reduce AFL contamination in crops have focused on the use of nonaflatoxigenic*A. flavus* isolates as biological control agents.  

Taxonomically, *A. flavus* belongs to the *Aspergillus* genus of the section *Flavi* [6, 7]. Molecular biology has offered several insights into the detection and genetic relationships of fungal isolates from their DNA sequences, taxonomy, population structure and the epidemiology associated with them [8]. Various molecular methods have been used for the detection of *Aspergillus* from environmental and clinical samples [9-11]. Targets for the genus level detection of *Aspergillus* have included the 18S rRNA gene, mitochondrial DNA, the intergenic spacer region, and the internal transcribed spacer (ITS) regions. Ribosomal RNA (rRNA) genes in ribosomal DNA possess characteristics that are suitable for the detection of pathogens at the species level [12]. These rDNA sequences are highly stable and exhibit a mosaic of conserved and diverse regions within the genome [13]. They also occur in multiple copies with up to 200 copies per haploid genome arranged in tandem repeats [8], each consisting of the 18S small subunit (SSU), the 5.8S, and the 28S large subunit (LSU) genes. ITS primers 1 and 4 have been used to amplify the entire 5.8S rRNA gene, both ITS regions I and II, and a portion of the 18S small-subunit rRNA gene. 

In recent years, there has been vast progress in the development of molecular biology tools and technologies [3,13-16]. Inter-simple sequence repeat (ISSR) is based on the amplification of regions(100-3000 bp) between inversely oriented, closely spaced microsatellites by primers (25-30 bp) consisting of several simple sequence repeats [17]. These primers anneal to simple-sequence repeats (microsatellites) that are abundant throughout the eukaryotic genome and evolve rapidly [16,18-19]. Prior knowledge of the DNA sequence of the genome to be analyzed is not required for primer design [20]. However, since there is a lot of diversity among fungi, primers that work for one may not work for another. Hence, ISSR primers need to be optimized for each species [21].

Microsatellite loci with di- to hexanucleotide repeats and 1000-bp flanking sequences were identified from the genome sequence of *A. flavus* NRRL3357 (http://www.aspergillusflavus.org/) using Tandem Repeats Finder version 4.00 [22]. Molecular typing of *A. flavus* using microsatellites yields multiple advantages such as high discriminatory power, high reproducibility and easy exchange of the results [14]. It is reported that the ISSR sequences as molecular markers that can lead to the detection of polymorphism, which is a new approach to study SSR distribution and frequency [23]. 

The specific aims of this work were to: 1. examine genetic relatedness among nonaflatoxigenic isolates of *A. flavus* belongIng to three Iranian pistachio, peanut and maize populations, and 2. assess polymorphisms among nonaflatoxigenic isolates of *A. flavus* using PCR amplification of ISSR molecular markers.

## MATERIALS AND METHODS

**Fungal strain: **Out of fifty-two nonaflatoxigenic isolates of *Aspergillus flavus *from three populations of *A. flavus* (peanut, maize and pistachio) isolated from different geographical origins (Guilan, Golestan, Ardebil, Fars, Kerman andSemnan provinces) and substrates (soil and kernel) (data not shown), 3, 7 and 5 representatives were randomly selected according to their vegetative compatibility groups (VCGs, IR1 to IR15) for microsatellite-primed PCR (MP-PCR) analysis (Table 1). Strain isolates were stored as spore suspensions in 20% glycerol at -20ºC. All strains had already been characterized for their aflatoxigenic ability after the mycelium collection yeast extract sucrose broths were analyzed by HPLC, to confirm AF production. This test is important because AF production is extremely dependent on growth conditions; it was, therefore, important to determine aflatoxigenic ability under current test conditions. Using specific primer pairs ITS1 and ITS4 as described previously [24],all*A. flavus* strains were identified and confirmed based on amplifications of internal transcribed spacers (ITS) of ribosomal DNA (rDNA) by polymerase chain reaction (PCR) combined with sequencing of the amplicons [25,26].

**DNA extraction: **Total DNA was extracted from themycelia of fungal isolates obtained from 7-day-old cultures grown in YES liquid media according to Prabha *et al.* (2012) with minor modifications [27]. Briefly, mycelia were collected by vacuum filtration, ground into a fine powder in liquid N_2_ and stored at -20°C. The frozen powder was then suspended in a 500 μl TES buffer (200 mM Tris-HCl, pH 7.5; 25 mM EDTA and 250 mM NaCl and 0.5% SDS), vortexed for 5 sec and incubated at 65 °C for 10 min. The reaction mixture was centrifuged at 13,000 rpm for 1 min and DNA was extracted with phenol/chloroform (1:1). DNA was then precipitated in 300 μl of cold iso-propanol and incubated for 30 min at –20°C and recovered by centrifugation at 13,000 rpm for 5 min.Afterwards, the pellet was washed with 70% cold ethanol and dried for 15 min at 37°C. Finally, the isolated DNA was resuspended in 50 μl of sterile distilled water and stored at -20°C. DNA concentration was measured spectrophotometrically with a NanoDrop Spectrophotomer ND-1000. DNA quality was also examined by running on 1.2% gel agarose for 75 min at 80 V, after which the gel was exposed to UV light. The presence of a highly resolved high molecular weight band and absence of smear confirmed the good quality of DNA.

**Molecular** **identification** **of** ***Aspergillus*** ***flavus*** : Identification of *A. flavus* using an Internal transcribed spacer (ITS) was conducted.Primer pairs (ITS1 and ITS4, Table 2) were derived from the ITS1-5.8S-ITS4 region [24]. PCR amplification was carried out in a 25 µl reaction mixture (Table 3) in a Biometra Thermal Cycler (T1 thermocycler; Biometra, Göttingen, Germany). The PCR product was analyzed by electrophoresis in 1.2% agarose gel stained with DNA green viewer dye (greenGel stain,10 mg/ml) and visualized with the UVsolo TS gel documentation system (Biometra).

**Table 1:** Monospore isolates used to evaluate polymorphisms in nonaflatoxigenicisolates of *Aspergillus flavus*, substrate, vegetative compatibility group (VCG) and geographical origin

**Strain isolate**	**Substrate**	**VCG**	**Geographical origin**
IRP-049	Soil/pistachio orchard	IR1	Rafsangan/Kerman province
IRP-107	Soil/ pistachio orchard	IR2	Rafsangan/Kerman province
IRP-082	Soil/ pistachio orchard	IR3	Damghan/Semnan province
IRP-144	Soil/ pistachio orchard	IR4	Damghan/Semnan province
IRG-075	Soil/Peanut field	IR5	Minoodasht,Golestan province
IRG-129	Soil/ Peanut field	IR6	Astane-e Ashrafieh/Guilan province
IRM-074	Soil/Maize field	IR7	Darab/Fars province
IRM-193	Soil/ Maize field	IR8	Fasa/Fars province
IRM-014	Soil/ Maize field	IR9	Pars Abad/Ardebil province
IRM-211	Soil/ Maize field	IR10	Pars Abad/Ardebil province
IRP-179	Kernel/ pistachio orchard	IR11	Rafsangan/Kerman province
IRG-517	Kernel/ Peanut field	IR12	Astane-e Ashrafieh/Guilan province
IRM-031	Soil/ Maize field	IR13	Pars Abad/Ardebil province
IRM-041	Kernel/ Maize field	IR14	Darab/Fars province
IRM-081	Kernel/ Maize field	IR15	Darab/Fars province

**Table 2:** Primer, target gene, sequence and expected PCR product size

**Primers**	**Region**	**Primer sequences (5ʹ3ʹ )**	**Annealing temp. (°C)**	**PCR product** **Size (bp)**
ITS1 ITS4	ITS	TCCGTAGGTGAACCTGCGG TCCTCCGCTTATTGATATGC	58	600

**Table 3:** PCR reaction mixture for amplification of the *ITS* region

**Final concentrations (volume)**	**Reaction mixture**
1X (2.5µl)	PCR buffer
50 mM (1 µl)	MgCl_2_
10 pmol/µl (1.5 µl)	Primer
2.5 mM/l(2 µl)	dNTPs
5 U/µl	Taq DNA polymerase
20 ng/µl (2 µl)	Template DNA
14.3 µl	D.D.W
25 µl	Total

**Microsatellite-primed PCR and electrophoresis: **Two ISSR primers that included AFMPP and AFM13 and showed more polymorphisms in previous studies were used [11, 22, 28,29] (Table 4). The genomic DNA sample was amplified using ISSR primers in a 25 µl reaction mixture containing PCR Buffer 1X, 0.2 µM ISSR primers, 3 mM MgCl_2_, 1 unit Taq DNA polymerase and 50 ng of the template DNA sample. The PCR was carried out in a Biometra Thermal Cycler (T1 thermocycler; Biometra, Göttingen, Germany) with the following profile: initial heating at 93°C for 5 min, thirty cycles of denaturation at 93°C for 30 s, annealing at 45°C for 1 min, extension at 72°C for 1.5 min and a final extension period at 72°C for 5 min. The result of each amplification reaction was analyzed on 2% agarose gel in a TBE buffer 1X (pH 8) and run at 80 V.Amplified fragments were then visualized using an ultraviolet transilluminator(UVsolo TS gel documentation system, Biometra) and compared with a 100 bp DNA size marker (Fermentas).

**Table 4:** List of ISSR primer sequences and their annealing temperatures (T_a_)

**Primers**	**Repeat motifs**	**Primer sequences (5´ 3´)**	**T**_a_ ** (°C)**
AFMPP	(GACA)_4_	GACAGACAGACAGACA	30.7
AFM13	(GTG)_5_	GAGGGTGGCGGTTCT	47.4

**Data analysis: **The internal transcribed spacer (ITS) region, ITS 1–5.8S–ITS 2, from nonaflatoxigenic isolates of *A. flavus* were amplified, sequenced, and compared with the reference strain sequence in GenBank. Gel images from ISSR-PCR fingerprint patterns of genomic DNAs were analyzed using 1D DNA gel image analysis software (TotalLab v2, Nonlinear Dynamics, Newcastle upon Tyne, UK) and dendrograms were constructed. The allele size was calculated using Alpha Imagersoftware [30]. The ladder in which all alleles were absent was used as an outgroup for dendrogram rooting.

## RESULTS AND DISCUSSION

**Figure 1:**
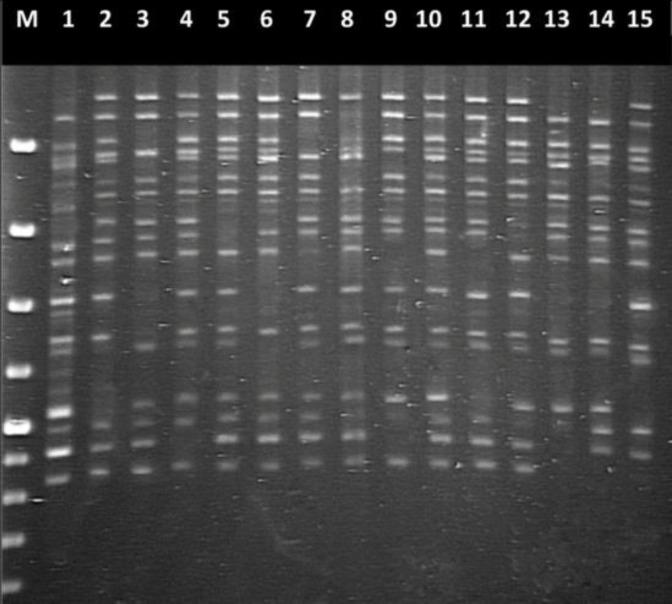
ISSR-PCR fingerprint pattern of genomic DNAs isolation from different nonaflatoxigenic isolates of *Aspergillus flavus* generated using primer ISSR M13. Lanes 1-15 were IRP49, IRP107, IRP179, IRP82, IRM41, IRM193, IRM74, IRP144, IRM81, IRM31, IRM14, IRM211, IRG129, IRG517, IRG75, respectively. M: molecular-weight marker (100 bp DNA ladder

**Figure 2:**
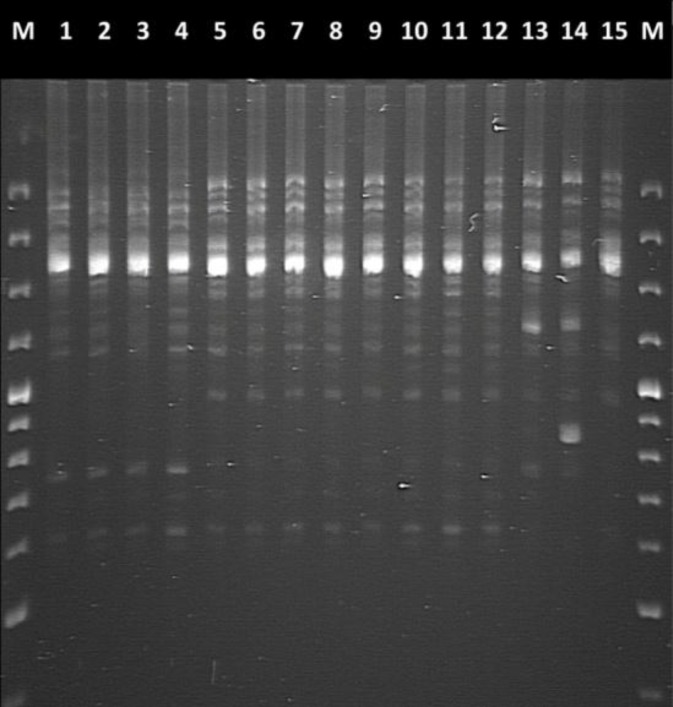
ISSR-PCR fingerprint pattern of genomic DNAs isolation from different nonaflatoxigenic isolates of *Aspergillus flavus* generated using primer ISSR MPP.Lanes 1-15 were IRP49, IRP107, IRP179, IRP82, IRM41, IRM193, IRM74, IRP144, IRM81, IRM31, IRM14, IRM211, IRG129, IRG517, IRG75, respectively. M: molecular-weight marker (100 bp DNA ladder

ITS amplicons from *A. flavus *strains were600 bpin size. Comparison of the reference strain and the GenBank sequence demonstrated that both *ITS 1* and *ITS 2* regions were needed for the accurate identification of*A. flavus*. ISSR profiles and allele sizes at ISSR markers AFM13 and AFMPP resulting from the analyses of Iranian nonaflatoxigenic strains of *A. flavus* are shown in Figures 1 and 2 and Tables 5 and 6, respectively. 

**Table 5:** Allele size of *Aspergillus flavus *at microsatellite marker AFM13 resulting from the analysis of nonaflatoxigenic isolates of *Aspergillus flavus* from Iran

**Lanes**	**1**	**2**	**3**	**4**	**5**	**6**	**7**	**8**	**9**	**10**	**11**	**12**	**13**	**14**	**15**
**Fragment size** (bp)	**-**	953	953	953	953	953	953	953	953	953	953	953	-	-	953
	928.5	928.5	928.5	928.5	928.5	928.5	928.5	-	928.5	928.5	928.5	928.5	928.5	928.5	928.5
	905	905	-	905	905	905	-	905	905	905	905	905	905	905	905
	895	895	895	895	895	895	-	895	895	895	895	895	895	895	895
	884	884	-	884	884	884	884	884	-	884	884	884	884	884	884
	863	863	863	863	863	863	863	-	863	863	863	863	863	-	863
	-	844.5	844.5	844.5	844.5	844.5	844.5	844.5	844.5	844.5	844.5	844.5	844.5	844.5	844.5
	834	834	834	834	834	834	834	834	834	834	834	834	834	-	-
	-	821	821	821	-	-	821	821	821	821	821	821	821	821	821
	805	805	805	805	-	805	805	805	805	805	805	-	805	805	805
	768.5	768.5	768.5	768.5	768.5	768.5	768.5	768.5	-	768.5	-	768.5	768.5	768.5	768.5
	747	747	-	747	747	-	747	747	747	747	747	747	-	-	-
	703	703	703	703	703	703	703	703	703	703	703	703	703	703	703
	669.5	669.5	-	669.5	669.5	669.5	669.5	669.5	669.5	669.5	669.5	669.5	669.5	669.5	669.5
	657	-	657	657	657	-	657	657	657	657	657	657	657	657	657
	638	-	-	-	-	-	-	-	-	-	-	-	-	-	-
	619	-	-	-	-	-	-	-	-	-	-	-	-	-	-
	592	-	-	-	-	-	-	-	-	-	-	-	-	-	-
	569	-	-		-	-	ـ	-	-	-	-	-	-	-	-
	515.5	-	515.5	515.5	515.5	515.5	515.5	515.5	515.5	515.5	-	515.5	515.5	515.5	-
	475	475	475	475	-	475	475	475	-	475	475	-	-	475	475
	425	425	425	-	425	425	425	425	-	425	425	425	-	425	425
	337.5	337.5	337.5	337.5	337.5	337.5	337.5	337.5	337.5	337.5	337.5	337.5	-	-	-

**Note:**Lanes 1-15 were IRP49, IRP107, IRP179, IRP82, IRM41, IRM193, IRM74, IRP144, IRM81, IRM31, IRM14, IRM211, IRG129, IRG517, IRG75, respectively.

**ISSR marker polymorphism: **The characteristics of ISSR marker polymorphisms are shown in Table 7. The percentages of polymorphic fragments for AFM13 and AFMPP were 95.7% and 66.7%, respectively. At the population level, the percentage of polymorphic bands from AFMPP ranged from 0% to 44.4%, and the average value was 25.9%. 

**Table 6:** Allele size of *Aspergillus flavus* at microsatellite marker AFMPP resulting from the analysis of nonaflatoxigenic isolates of *Aspergillus flavus* from Iran

**Lanes**	**1**	**2**	**3**	**4**	**5**	**6**	**7**	**8**	**9**	**10**	**11**	**12**	**13**	**14**	**15**
**Fragment size** **(bp)**	920	920	920	920	-	-	-	-	-	-	ـ	-	-	-	-
	-	-	-	-	900	900	900	900	900	900	900	900	900	900	900
	880	-	880	880	880	880	880	880	880	880	880	880	880	-	880
	843	843	843	843	843	843	843	843	843	843	843	843	843	843	843
	810	810	810	810	810	810	810	810	810	810	810	810	810	810	810
	778	778	778	778	778	778	778	778	778	778	778	778	778	778	778
	721	721	721	721	721	721	721	721	721	721	721	721	721	721	721
	710	710	710	710	710	710	710	710	710	710	710	710	710	710	710
	667	667	667	667	667	667	667	667	667	667	667	667	667	667	667
	644	644	-	644	644	644	644	644	644	644	644	644	644	644	-
	602	602	-	602	602	602	602	602	602	602	602	602	602	602	-
	563	563	-	563	563	563	563	563	563	563	563	563	563	563	563
	-	-	-	-	520	520	520	520	520	520	520	520	520	520	520
	-	-	-	-	-	-	-	-	-	-	-	-	-	500	-
	475	475	475	475	475	475	475	475	475	475	475	475	475	475	-
	410	410	410	410	410	410	410	410	410	410	410	410	410	-	-
	338	338	338	338	338	338	338	338	338	338	338	338	338	-	338
	312	312	312	312	312	312	312	312	312	312	312	312	312	-	312

**Note:**Lanes 1-15 were IRP49, IRP107, IRP179, IRP82, IRM41, IRM193, IRM74, IRP144, IRM81, IRM31, IRM14, IRM211, IRG129, IRG517, IRG75, respectively

**Table 7:** Characteristics of ISSR marker polymorphisms

**Primer sequence**	**No. of fragments**	**No. of polymorphic fragments**	**% polymorphic fragments**	**Product size** **range (bp)**
(GACA)_ 4_	18	12	66.7	312-920
(GTG)_ 5_	23	22	95.7	475-953

**Figure 3:**
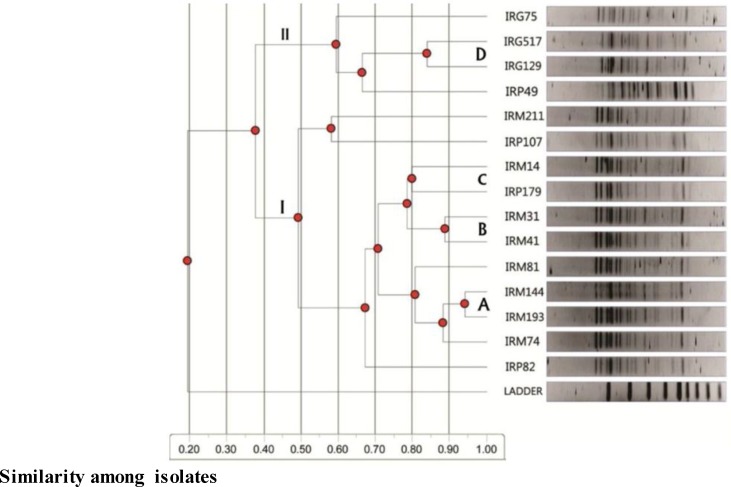
Dendrogram showing clustering of Iranian nonaflatoxigenic isolatesof *A. flavus*based on genetic similarities in PCR reactions using ISSR primer AFM13. The two main groups formed are shown under I and II. Strain isolates with highest genetic similarities (more than 80%), are indicated by the letters A, B, C and D.(Ladder=Outgroup).

**Figure 4:**
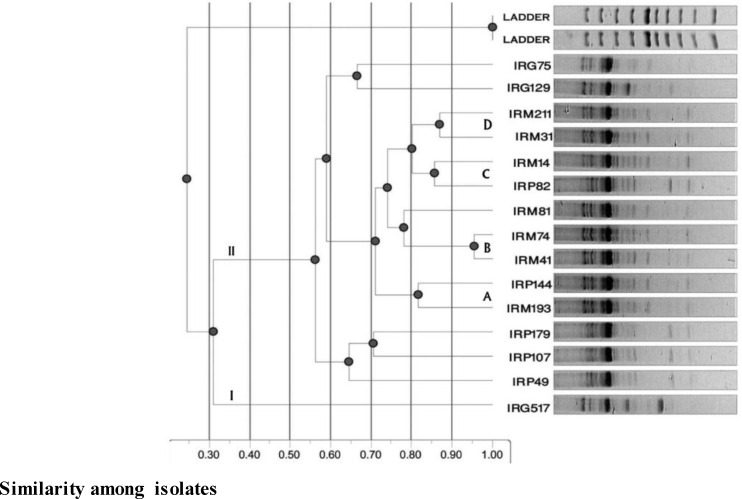
Dendrogram showing clustering of Iranian nonaflatoxigenic isolates of *A. flavus*based on genetic similarities in PCR reactions using ISSR primer AFMPP. The two main groups formed are shown under I and II. Strain isolates with highest genetic similarities (more than 80%), are indicated by the letters A, B, C and D.(Ladder=Outgroup

**Phylogenetic analysis: **Polymorphic fragments were used for the statistical interpretation of phylogenetic relations (Total Lab 120, UVP soft). Figures 3 and 4 show the dendrograms constructed using cluster analysis. Genetic similarities (x-axis) are expressed as 0–1. AFM13 and AFMPP initially split all fifteen nonaflatoxigenic isolates of *A. flavus* into two main groups (I and II) at 3.8% and 3.1% genetic similarities, respectively (Figures 3 and 4).

The larger primer groups AFM13 and AFMPP comprised two and three subgroups, respectively, each spliting further into six smaller groups (I-VI) containing one to four *A. flavus* strain isolate(s) (Figures 3 and 4). Otherwise, using ISSR primers AFM13 and AFMPP, the fifteen strain isolates belonging to three populations of Iranian nonaflatoxigenic isolates of *A. flavus* from pistachio, maize and peanut were separated and placed into six distinct clusters based on genetic similarities (Figures 3 and 4).

In the present study, a method to identify *A. flavus *strain isolateswas developed using the 18S and 28S rRNA genes for primer binding sites. rDNA has been utilized by many investigators for species determination in a wide variety of yeasts and fungi [31-33]. In this research, 15 nonaflatoxigenic strains of *A. flavus *belonging to different substrates, geographical regions and VCGs were analyzed to determine the degree of polymorphism. The ISSR marker was identified by PCR amplification of DNA using primer pairs composed of microsatellite sequences that may be anchored at the 3'or 5' end of 2 to 4 arbitrary and often degenerate nucleotides [34,35]. The results indicated that to amplify ISSR sequences in DNA extracted from nonaflatoxigenic isolatesof *A. flavus*,ISSR primers AFMPP and AFM13 produced positive results from the PCR trials. ISSR, which is a dominant marker, has greaterrobustness in repeatabilityand high variability [36]. The two ISSR primers (AFMPP and AFM13) produced a series of discrete bands of different intensities at annealing temperatures 30.7°Cand 47.4°C. Several isolates had similar banding patterns such as thosein lanes 1 and 4 (strain isolates IRP49 and IRP82) and lanes 5 to 13 (strain isolates IRM41, IRM193, IRM74, IRP144, IRM81, IRM31, IRM14, IRM211, IRG129) in the AFMPP profile. 

Altogether, the ISSR primers AFM13 and AFMPP generated 232 and 135 polymorphic bands ranging from 337 bp to 953 bp and 312 bp to 920 bp across fifteen strain isolates, respectively. Of the 23 and 18 ISSR discernible bands from primers AFM13 and AFMPP, 22 and 12 were polymorphic, respectively.

The Iranian nonaflatoxigenic isolates exhibited a high level of polymorphism, which was reflected in the number and percentage of polymorphic loci. Because of its simple technology and high level of polymorphism, microsatellite-primed PCR has been widely used for population genetic studies [22,37-39]. They produce different numbers of DNA fragments, depending on their simple sequence repeat motifs. In the current study, it was found that the ISSR AFM13 (GTG)_5_ tested was more polymorphic among our nonaflatoxigenic isolates. ISSR analysis aims at studying the polymorphism of highly repetitive genome regions [39,40]. The percentage of polymorphic bands from AFM13 ranged from 23.1% to 69.9%, and the average value was 45.5%. Usually, ISSR primers based on di- and tri- nucleotide repeats reveal high polymorphisms [41,42] which was also found to be true for the present study. Hatti *et al.* (2010) reported an average of 9.33 polymorphic bands per ISSR primer [43]. In contrast, Batista *et al.* (2008) showed high genetic variability among strains of *A. flavus* and other species of the *A. flavus* group by using the ISSR marker [28]. They showed that the (GACA)_4_ primer yielded a higher polymorphism as compared to (GTG)_5_. 

In our study, some polymorphic bands appeared more than once across the different strain isolates. Primers based on a repeat sequence and the resulting PCR reaction amplify the sequence between two ISSRs, yielding a multilocus marker system [43]. The dendrogram analysis for AFM13 showed that cluster II was comprised of IRM74, IRM193, IRM144 and IRM81isolates, while cluster III contained IRM41, IRM31, IRP179 and IRM14 isolates. Clusters IV and V possessed IRP107, IRM211 and IRP49, IRG129, IRG75 isolates, respectively. Strain isolates IRP82 and IRG517 grouped into clusters I and VI, respectively, showed their separate identities in comparison with other isolates. Although ISSRs are mostly random-type markers, they are thought to be highly useful for genetic diversity and phylogenetic studies [18]. 

For the ISSR primer AFMPP, cluster II comprised of IRP49, IRP107 and IRP179, while cluster III contained IRM193 and IRP144 isolates. Clusters IV and V possessed IRM41, IRM74, IRM81 and IRP82, IRM14, IRM31, IRM211 isolates, respectively. Strain isolates IRG517 and IRG75, IRG129 grouped into clusters I and VI, respectively, showed their separate identities in comparison with other isolates. Therefore, it can be concluded that ISSR markers could be used to study population structure among*A. flavus *and related species [12,28,29,44].

The similarity for maize (IRM193, IRM144, IRP41, IRM31, IRM14 and IRP179) and peanut strain isolates (IRG517 and IRG129) reached over 80% for the ISSR primer AFM13. Likewise, the similarity for maize (IRM193, IRP144, IRM41, IRM74 and IRM14) and peanut strain isolates (IRP179, IRG517 and IRG129) reached over 80% for the ISSR primer AFMPP. According to previous studies, ISSR markers have been used to determine similarity and dissimilarity between aflatoxigenic and nonaflatoxigenic isolates of *A. flavus *[43].

Similar to Yin* et al.* (2009) who showed that the toxigenic and atoxigenic isolates of *A. flavus*, collected from peanut fields, were not clustered based on their regions, ability of aflatoxin and sclerotial production [33], in the present study, the analysis of microsatellite-primed PCR data showed that Iranian nonaflatoxigenic isolates of the *A. flavus* were also not clustered based on their geographical origins and substrates. To the researchers’ knowledge, this is the first study of population analysis of nonaflatoxigenic isolates of *A. flavus* based on microsatellite-primed PCR in Iran.

Biological variability and the management of genetic variation within a species is a commonly recognized value in natural resources administration. Two primers, AFM13 and AFMPP gave reproducible banding profiles for most Iranian nonaflatoxigenic isolates of *A. flavus* tested. In this study, the ISSRs exposed significant numbers of polymorphisms, providing indication of *A. flavus* variability. Each of the two ISSR primers revealed a relatively high intra-species variability among the *A. flavus *isolates with considerable variation in morphological features. ISSR has an advantage over randomly amplified polymorphic DNA (RAPD) because its primers are longer, allowing for higher annealing temperatures that apparently provide a higher reproducibility of fragments than RAPD. Cluster analysis of the ISSR data divided the isolates of *A. flavus *to groups. The different subgroups formed by each primer were indicative of intra-species variability. The ecological nichemay be used to explain how the several groups of *A. flavus* strain isolates were formed by the ISSR primers.

The varying similarity ranges within strain isolates of the *A. flavus* could also be a result of isolates that share a host range and/or ecological niche. Population genetics data can provide valuable information, often unattainable via other approaches, for monitoring species of management, conservation and ecological interest. Our experiments have demonstrated that ISSR analysis is a powerful tool for the identification of polymorphisms in Iranian nonaflatoxigenic isolates of *A. flavus*. Whilst this technique gives useful information, several other ISSR primers are needed for more reliable results.

## Notes

**Acknowledgments:**Funding was provided by Ferdowsi University of Mashhad, Iran. We deeply appreciate the respected reviewers for their valuable suggestions to improve the manuscript.
